# Neuropsychological deficits in patients with cognitive complaints after COVID‐19

**DOI:** 10.1002/brb3.2508

**Published:** 2022-02-08

**Authors:** Carmen García‐Sánchez, Marco Calabria, Nicholas Grunden, Catalina Pons, Juan Antonio Arroyo, Beatriz Gómez‐Anson, Alberto Lleó, Daniel Alcolea, Roberto Belvís, Noemí Morollón, Isabel Mur, Virginia Pomar, Pere Domingo

**Affiliations:** ^1^ Neuropsychology Unit Neurology Department Hospital de la Santa Creu i Sant Pau Barcelona Spain; ^2^ Faculty of Health Sciences Universitat Oberta de Catalunya Barcelona Spain; ^3^ Department of Psychology Concordia University Montreal Canada; ^4^ Facultat de Psicologia Ciències de l'Educació i l'Esport Blanquerna Universitat Ramon Llull Barcelona Spain; ^5^ Internal Medicine Department Hospital de la Santa Creu i Sant Pau Barcelona Spain; ^6^ Neurodiagnostic Department Hospital de la Santa Creu i Sant Pau Barcelona Spain; ^7^ Memory Unit Neurology Department Hospital de la Santa Creu i Sant Pau Barcelona Spain; ^8^ Headache Unit Neurology Department Hospital de la Santa Creu i Sant Pau Barcelona Spain; ^9^ Infectious Disease Unit Hospital de la Santa Creu i Sant Pau Barcelona Spain

**Keywords:** cognition, cognitive complaints, neuropsychology, post‐COVID‐19

## Abstract

**Background:**

While much of the scientific focus thus far has been on cognitive sequelae in patients with severe COVID‐19, subjective cognitive complaints are being reported across the spectrum of disease severity, with recent studies beginning to corroborate patients’ perceived deficits. In response to this, the aims of this study were to (1) explore the frequency of impaired performance across cognitive domains in post‐COVID patients with subjective complaints and (2) uncover whether impairment existed within a single domain or across multiple.

**Methods:**

Sixty‐three patients with subjective cognitive complaints post‐COVID were assessed with a comprehensive protocol consisting of various neuropsychological tests and mood measures. Cognitive test performance was transformed into T scores and classified based on recommended guidelines. After performing a principal component analysis to define cognitive domain factors, distributions of test scores within and across domains were analyzed.

**Results:**

Results revealed pervasive impact on attention abilities, both as the singularly affected domain (19% of single‐domain impairment) as well as coupled with decreased performance in executive functions, learning, and long‐term memory. These salient attentional and associated executive deficits were largely unrelated to clinical factors such as hospitalization, disease duration, biomarkers, or affective measures.

**Discussion:**

These findings stress the importance of comprehensive evaluation and intervention to address cognitive sequelae in post‐COVID patients of varying disease courses, not just those who were hospitalized or experienced severe symptoms. Future studies should investigate to what extent these cognitive abilities are recuperated over time as well as employ neuroimaging techniques to uncover underlying mechanisms of neural damage.

## Graphical abstract

This figure helps to visualize the frequency of deficits by cognitive domain. Additionally, it allows the reader to see what percentage of patients showed single‐domain versus multi‐domain cognitive impairment.

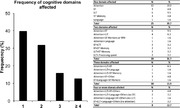

## 1 INTRODUCTION

After more than 1 year since the beginning of the pandemic, we have consistent evidence that the disease caused by SARS‐CoV‐2 infection, COVID‐19, is a multisystemic syndrome (Jiang et al., 2020). This coronavirus has been shown to cause multi‐organ dysfunction, affecting not only the lungs but also the heart, kidneys, gut, liver (Batlle et al., 2020; Long et al., 2020; Terpos et al., 2020), and the brain (Harapan & Yoo, 2021; Stein et al., 2021; Wei et al., 2020). Even when relatively little was known about COVID‐19 symptomatology, reports of neurological complications associated with SARS‐CoV‐2 were published (Moriguchi et al., 2020; Ye et al., 2020); subsequently, the impact of this coronavirus on the brain has been a topic of great interest (Leonardi et al., 2020).

Our understanding of COVID‐19's effect on the central nervous system (CNS) is still limited. However, researchers have proposed indirect pathways that may cause brain damage through inflammatory changes, coagulopathy, and vascular endothelial dysfunction (Boldrini et al., 2021; Marshall, 2020; Peiris et al., 2021; Solomon, 2021). Additionally, brain imaging studies have uncovered altered cerebral glucose metabolism in the subacute stage of COVID‐19, predominantly at frontoparietal level (Hosp et al., 2021). In other cortical areas such as the superior temporal, precentral, and lateral occipital cortices (Parsons et al., 2021), as well as medial temporal structures and hippocampus (Ladopoulos et al., 2021), lesions due to ischemia, acute white matter abnormalities, encephalopathic changes, or intracranial hemorrhages associated with COVID‐19 (Gulko et al., 2020; Ladopoulos et al., 2021; Mahammedi et al., 2021) have been found.

Although the frequency and severity of neurological symptoms associated with COVID‐19 may vary to some degree according to the severity of neuroinflammation and other medical conditions (Nordvig et al., 2021; Yassin et al., 2021), recent findings show that COVID‐19 survivors with a wide range of disease courses, ranging from asymptomatic (Amalakanti et al., 2021) to those with severe symptoms (Negrini et al., 2021; Whiteside et al., 2021), may present with both short‐ and long‐term cognitive deficits. In a recent review, Alnefeesi et al. (2021) assessed seven studies and concluded that COVID‐19 predominantly affects long‐term memory and executive functions.

To date, epidemiologic studies with large samples of patients have used screening tests for global cognition or qualitative assessments of mental status (García‐Azorín et al., 2021; Raman et al., 2021; Taquet et al., 2021). While the findings from these studies serve as useful evidence in revealing general cognitive impact associated with COVID‐19, results from screening tests cannot be used to address the question of specificity in cognitive deficits. Other studies with smaller sample sizes have begun to apply more comprehensive neuropsychological evaluations, providing a more detailed picture of deficits in the weeks and months following infection and/or hospital discharge. Short‐term deficits were found in attention (Almeria et al., 2020; Zhou et al., 2020), in long‐term memory, attention and executive functions (Almeria et al., 2020), and in inhibitory control (Ortelli et al., 2021). Considering more long‐term effects, Ferrucci et al. (2021) reported evidence of long‐term memory deficits in COVID‐19 patients 5 months after hospitalization and Mazza et al. (2021) found that half of their COVID‐19 patient sample demonstrated executive function deficits and 30% of them showed impairments in information processing, verbal fluency, and working memory at a 3‐month follow‐up. In contrast to these findings, Mattioli et al. (2021) found no evidence of any cognitive impairment in patients with COVID‐19 compared to a group of control participants 4 months after SARS‐CoV‐2 infection.

It is also important to define the nature of deficits in terms of single versus multiple‐domain impact. In making this distinction, the existence of deficits within or across domains can begin to indicate how widespread the impacts on underlying cognitive mechanisms are. As research from neuroimaging studies in COVID‐19 survivors has thus far shown a heterogeneous picture of brain damage, the presence of multiple‐domain deficits would further confirm the diverse effects of COVID‐19 on the brain. Within the current literature, one line of evidence is indicating that medial temporal structures and hippocampus (Ladopoulos et al., 2021) are affected; given this, memory‐related deficits would then be expected, mainly for the consolidation of new information. Indeed, findings from studies that investigated neuropsychological impairments in COVID‐19 reveal long‐term memory deficits (Almeria et al., 2020; Ferrucci et al., 2021). Other studies have found changes in metabolism at frontoparietal level (Hosp et al., 2021), which would suggest potential impairments in the attentional networks. Linking this to neuropsychological findings, deficits of attention and executive functions are indeed reported in many studies (Almeria et al., 2020; Ortelli et al., 2021; Zhou et al., 2020). With these two emerging links between brain and behavior, it is crucial to know the extent to which patients have both memory and executive function deficits in order to determine whether they could be explained by the same processes.

In the present study, we focus on investigating a sample of outpatients who reported subjective cognitive complaints after SARS‐CoV‐2 infection. Using tests spanning across different cognitive domains, the study sought to determine which cognitive abilities are most affected in this population. Addressing remaining uncertainties outlined above, we had two aims for this study: (1) to analyze the frequency of deficits for specific cognitive domains and (2) to discern the frequency of single‐ and multiple‐domain impairments and to understand which combinations of deficits were a specific feature of post‐COVID‐19 cognitive impairment. Additionally, we also investigated whether certain clinical factors were associated with cognitive impairment.

## 2 METHODS

### 2.1 Participants

The study included 84 consecutive patients evaluated from July 5, 2020 to May 26, 2021 at the Neuropsychology Unit of the Hospital de la Santa Creu i Sant Pau (HSCSP) in Barcelona (Spain) with subjective cognitive complaints after SARS‐CoV‐2 infection. The inclusion criteria for this study were: (a) having had COVID‐19 symptoms and confirmed positive for SARS‐CoV‐2 via polymerase chain reaction (PCR) and/or serology (anti‐SARS‐CoV2 IgM or IgG); (b) referred for neuropsychological assessment after reporting subjective cognitive complaints; and (c) were 18+ years old. The exclusion criterion was documented medical history of neurological or psychiatric conditions before the infection. After reviewing their medical records, 21 were excluded as probable COVID (negative results when tested for SARS‐CoV‐2 or not tested at all). The final sample included 63 participants.

### 2.2 Neuropsychological assessment

The neuropsychological study was carried out on patients who presented subjective complaints after having suffered COVID‐19. The tests were administered by an expert neuropsychologist at HSCSP over two sessions, with a maximum interval between these sessions of 10 days.

A comprehensive neuropsychological assessment included multiple tests for each of the following cognitive domains: general cognitive status, attention, short‐ and long‐term memory, language, processing speed, visuoperceptual–visuoconstructive functions, and executive functions. Specifically, the assessment included the following tests: MoCA, CPT‐II, RAVLT, ROCFT, Digit Span Forward and Backward, BNT, Block Design, Coding, Symbol Search, TMT, Stroop, verbal fluency tasks, and the 15‐Objects Test (see Supporting Information for a complete list with full test names, descriptions, and normative data). In addition to cognitive measures, patients were administered the Hospital Anxiety and Depression Scale (HADS; Zigmond & Snaith, 1983) as a brief measure for anxiety and depression levels in patients.

## 3 ANALYSIS

Individual raw scores from each test were transformed into T scores according to the normative data available. T scores were then transformed into percentiles (Pc) and classified according to the ranges proposed by American Academy of Clinical Neuropsychology (Guilmette et al., 2020). The classification is the following: exceptionally high score: Pc > 98; above average score: Pc 91–97; high average score: Pc 75–90; average score Pc: 25–74; low average score: Pc 9–24; below average score: Pc 2–8; exceptionally low score: Pc < 2. Then, we classified individual test scores for each test according to these three main categories: (a) below average score and exceptionally low score (Pc < 8), low average score (Pc: 9–24), and average or above (Pc > 25).

**FIGURE 1:**
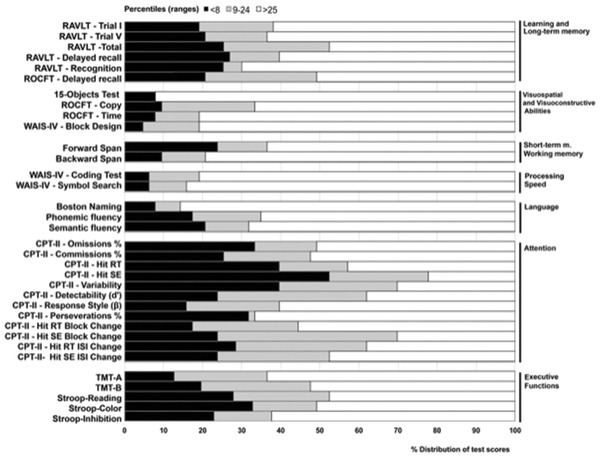
Distribution of test scores according to classification by Guilmette et al. (2020): combined below average score and exceptionally low score (Pc < 8), low average score (Pc: 9–24), and average or above (Pc > 25)

First, we performed an analysis that aimed to describe the frequency of presence or absence of cognitive deficits according to the neuropsychological test scores as a function of the three categories, such as being a below average score/exceptionally low score, low average score, and average or above (Pc > 25). The distribution of test scores was analyzed by grouping them in cognitive domains. To define said cognitive domains, we ran a principal component analysis (PCA) that included the entire test scores (see Table 1 and Figure 1) from the neuropsychological assessment.

**TABLE 1:** Distribution of test scores according to classification by Guilmette et al. (2020): Combined below average score and exceptionally low score (Pc < 8), low average score (Pc: 9–24), and average or above (Pc > 25)

	Distribution of test scores				% Distribution of test scores			
Percentiles	<8	9–24	>25	Total	<8	9–24	>25	<24
**Learning and long‐term memory (L + LTM)**								
RAVLT – Trial I	12	12	39	63	19.05	19.05	61.90	38.10
RAVLT – Trial V	13	10	40	63	20.63	15.87	63.49	36.51
RAVLT – Total	16	17	30	63	25.40	26.98	47.62	52.38
RAVLT – Delayed recall	17	8	38	63	26.98	12.70	60.32	39.68
RAVLT – Recognition	16	3	44	63	18.33	5.00	76.67	23.33
ROCFT – Delayed recall	13	18	32	63	20.63	28.57	50.79	49.21
**Visuospatial and visuoconstructive abilities (VVA)**								
15‐Objects test	5	–	58	63	7.94	–	92.06	7.94
ROCFT – Copy	6	15	42	63	9.52	23.81	66.67	33.33
ROCFT – Time	5	7	51	63	7.94	11.11	80.95	19.05
WAIS‐IV – Block design	3	9	51	63	4.76	14.29	80.95	19.05
**Short‐term and working memory (ST/WM)**								
Forward span	15	8	40	63	23.81	12.70	63.49	36.51
Backward span	6	7	50	63	9.52	11.11	79.37	20.63
**Processing speed**								
WAIS‐IV – Coding test	4	8	51	63	6.35	12.70	80.95	19.05
WAIS‐IV – Symbol search	4	6	53	63	6.35	9.52	84.13	15.87
**Language**								
Boston naming	5	4	54	63	7.94	6.35	85.71	14.29
Phonemic fluency	11	11	41	63	17.46	17.46	65.08	34.92
Semantic fluency	13	7	43	63	20.63	11.11	68.25	31.75
**Attention**								
CPT‐II – Omissions %	21	10	32	63	33.33	15.87	50.79	49.21
CPT‐II – Commissions %	16	14	33	63	25.40	22.22	52.38	47.62
CPT‐II – Hit RT	25	11	27	63	39.68	17.46	42.86	57.14
CPT‐II – Hit SE	33	16	14	63	52.38	25.40	22.22	77.78
CPT‐II – Variability	25	19	19	63	39.68	30.16	30.16	69.84
CPT‐II – Detectability (d')	15	24	24	63	23.81	38.10	38.10	61.90
CPT‐II – Response Style (β)	10	15	38	63	15.87	23.81	60.32	39.68
CPT‐II – Perseverations %	20	1	42	63	31.75	1.59	66.67	33.33
CPT‐II – Hit RT block change	11	17	35	63	17.46	26.98	55.56	44.44
CPT‐II – Hit SE block change	15	29	19	63	23.81	46.03	30.16	69.84
CPT‐II – Hit RT ISI change	18	21	24	63	28.57	33.33	38.10	61.90
CPT‐II – Hit SE ISI change	15	18	30	63	23.81	28.57	47.62	52.38
**Executive functioning (EF)**								
TMT‐A	8	15	40	63	12.70	23.81	63.49	36.51
TMT‐B	12	17	32	61	19.67	27.87	52.46	47.54
Stroop – Color	20	10	31	61	32.79	16.39	50.82	49.18
Stroop – Inhibition	14	9	38	61	22.95	14.75	62.30	37.70

*Abbreviations*: CPT‐II, Conners' continuous performance test II; RAVLT, Rey auditory verbal learning test; ROCFT, Rey–Osterrieth complex figure test; TMT‐A, TMT‐B, trail making test (part A and B); WAIS‐IV, Wechsler adult intelligence scale – fourth edition.

Second, we analyzed the frequency of cognitive deficits in patients by classifying neuropsychological tests according to the domains defined by the PCA. A cognitive domain was classified as affected if matched one of these criteria: (a) at least 50% of the scores with a percentile below 8 for the tests having multiples scores; (b) at least 50% of the test scores with a percentile below 8 for the tests having single scores; (c) at least 30% of the test scores with a percentile below 8 and 30% of the test scores with a percentile between 9 and 24.

The aim of this analysis was to see whether the impact of SARS‐CoV‐2 infection on cognition multi‐domain or not. Additionally, we aimed to explore the most frequent associations of cognitive deficits. Correlational analyses were performed between the mean T scores of the more frequently affected cognitive domains. Mean T scores were calculated by averaging the T scores of the test within the same cognitive domain.

Finally, we performed further analyses to explore the effect of hospitalization, disease duration, biomarkers, and affective scores on cognition.

## 4 RESULTS

### 4.1 Patient sample characteristics

This final sample was composed of 22 males (35%) and 41 females (63%), with a mean age of 51.1 years (SD = 12.5; range: 22–78) and mean level of education of 14.4 years (SD = 3.1). The time between the diagnosis of COVID‐19 and the neuropsychological assessment was an average of 187 days (SD = 99). According to the World Health Organization (2021), this duration is compatible with the definition of “post‐COVID” with the presence of clinical symptoms after a probable or confirmed SARS‐CoV‐2 infection 3 months after the onset of COVID‐19 (World Health Organization, 2021).

Thirty‐three patients (52.4%) were hospitalized for an average 20 days (SD = 16.9) and 15 of them entered the intensive care unit (ICU) (M = 13.6 days; SD = 10.0). Non‐hospitalized patients were younger (M = 46.7; SD = 11.8) than hospitalized patients (M = 55.9; SD = 11.9; *p* < .001), but with the same mean year of education (non‐hospitalized: M = 14.5, SD = 2.9; hospitalized: M = 14.2; SD = 3.3; *p* = .73) and with no group difference for the days elapsed between diagnosis and testing (non‐hospitalized: M = 177.5, SD = 106.1; hospitalized: M = 198.6, SD = 92.3; *p* = .40).

Fifteen patients were treated with hydroxychloroquine (400 mg/day) during a mean duration of 5 days and five of them were also treated with Tocilizumab (400 or 600 mg). Nine patients were treated with corticosteroids for a mean duration of 6.3 days (various doses).

### 4.2 Distribution of test scores

Since we used several tests with many scores, we decided to reduce the number of variables by grouping them into cognitive domains. This allowed us to better describe our results in terms of cognitive processes that are affected or spared in our sample.

To define the cognitive domains implicated in this study, we ran a PCA that included the percentiles from all tests used in the neuropsychological assessment, excluding the MoCA due to its spanning of multiple cognitive domains as a screening tool. To correct for the common covariance of the variables, a rotation with the direct oblimin method was applied to the factor matrix. After rotation, the result of the PCA suggested that the best factorial solution saturated into five main factors, as it explained 80.3% of the variance. The factors from the PCA were:

1. Learning and Long‐Term Memory (L + LTM), including all scores on the RAVLT (Delayed recall: 0.88; Trial V: 0.84; Recognition: 0.79; Total: 0.78; Trial I: 0.47);
2. Visuospatial and Visuoconstructive Abilities (VVA), including scores on the Block Design Test (0.64), ROCFT – Copy (0.48) and 15‐Objects Test (0.71).
3. Language, including scores on the semantic (0.77) and phonological (0.43) fluency tests, as well as the Boston naming test (0.46).
4. Attention, including sustained attention scores on the CPT‐II (Omissions: 0.91; Commissions: 0.87; Variability: 0.83; Hit RT: 0.81; Detectability: 0.81; Perseveration: 0.77; Hit SE: 0.62; Hit RT ISI Change: 0.46);
5. Executive Functioning (EF), including scores on the Stroop task (Reading: 0.84; Color: 0.80; Inhibition: 0.79) and Trail Making Tests (A: 0.65; B: 0.762).

The scores from the forward and backward digit spans showed low loadings and were not clearly associated with any of the five main factors, so we grouped them under the category “Short‐Term and Working Memory” (ST/WM). WAIS‐IV Coding Test and WAIS‐IV Symbol Search were not associated with the other main factors from the PCA, so we grouped them under the category “Processing Speed..”

Finally, as some of the scores on the CPT‐II remained outside the main factor “Attention” but were from the same test, they were included in the same factor for the purpose of the analyses. The same reasoning was applied to the score on ROCFT – Delayed recall, which was included in the L + LTM factor.

As shown in Table 1, the amount of test scores that were suggestive of cognitive deficits (below average score and exceptionally low score, Pc < 8) varied across domains. Specifically, the share of abnormal test scores ranged from 18.33% to 26.98% depending on the test in the L + LTM factor, from 4.76% to 9.52% for VVA, was 9.52% for short‐term memory and 23.81% for working memory within the ST/WM factor, 6.35% for Processing Speed, ranged from 7.94% to 20.63% for Language, from 15.87% to 52.38% for Attention, and from 12.70% to 32.79% for EF. If we include low average scores (Pc: 9–24), an increase of cognitive domains affected can be observed, especially for L +LTM (23.33–52.38%), Attention (33.33–77.78%), and EF (36.51–52.46%).

### 4.3 Proportions of patients with cognitive deficits by domains

**FIGURE 2:**
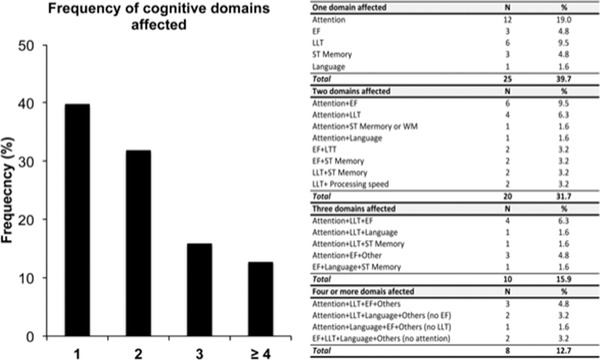
Frequency of affected cognitive domains and distributions of deficits for single‐ and multi‐domain cognitive impairment

Multiple‐domain impairment (60.3%) was more frequent than impairment in only one domain (39.7%) (*χ* ^2^(1) = 5.36, *p* = .02). Attention deficits were the most frequent types of deficits in patients with single‐domain impairment (19.0%), significantly exceeding deficits in EF (*p* = .01), ST/WM (*p* = .001), and Language (*p* < .001). Furthermore, attention was the cognitive domain that was most frequently impaired in conjunction with other domains in patients with multiple‐domain impairment, especially with L + LTM and EF (see Figure 2).

**FIGURE 3:**
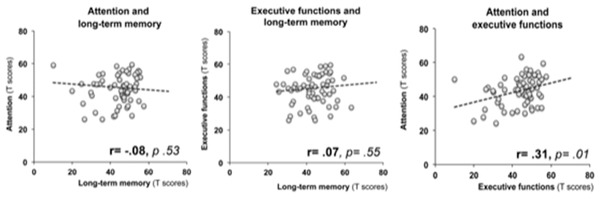
Correlation between attention, long‐term memory, and executive functions

To see whether the performance in tests assessing attention, long‐term memory, and executive functions were explained by overlapping deficits, we performed correlational analyses using the composite scores derived from the T scores for the three domains. Only executive functions and attention were significantly correlated (*r* = .31, *p* = .01) (see Figure 3).

### 4.4 Hospitalization, disease duration, biomarkers, and affective scores

We performed further analyses to explore the effect of hospitalization, disease duration, and biomarkers on cognition.

#### 4.4.1 Hospitalization

**FIGURE 4:**
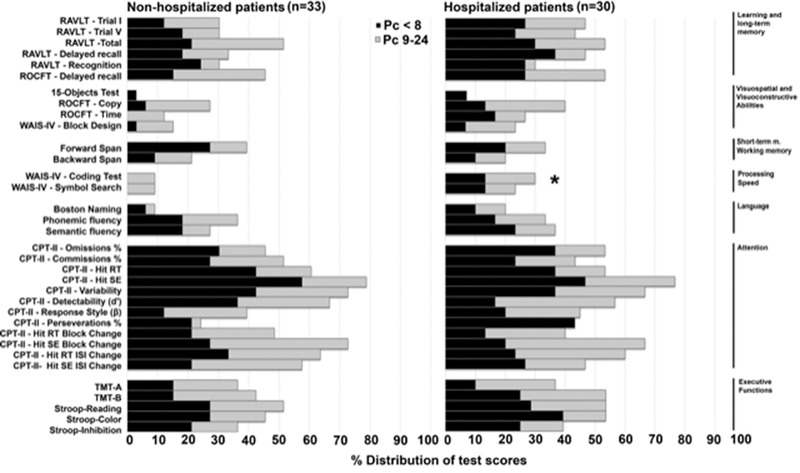
Distribution of test scores between hospitalized and non‐hospitalized patients according to classification by Guilmette et al. (2020): combined below average score and exceptionally low score (Pc < 8) versus low average score (Pc: 9–24). Asterisk indicates a significant difference in test performance between groups

Hospitalized patients had lower MoCA scores (M = 15.8; SD = 3.8) than non‐hospitalized ones (M = 17.8; SD = 2.5) (F (1, 61)) = 6.66; *p* < .05; age introduced as a covariate). To explore in more detail the cognitive deficits that may be associated with hospitalization, we performed group comparisons of the distributions for average score and exceptionally low score (Pc < 8), low average score (Pc: 9–24), and both combined (Pc: 0–24). The only significant group difference was found when the percentiles were combined for the Coding test (*χ* ^2^(1) = 4.45, *p* = .03), suggesting that hospitalization is associated with decreased performance in processing speed (see Figure 4).

#### 4.4.2 Disease duration

Correlations between individual disease durations and the composite scores for each cognitive domain were not significant (Attention: *r* = 0.20, *p* = .25; L + LTM: *r* = −0.06, *p* = .72; EF: *r* = −0.07, *p* = .71; Language: *r* = −0.18, *p* = .32; Processing Speed: *r* < 0.01, *p* = .95; VVA: *r* = −0.01, *p* = .91), suggesting that there is no effect of this variable on the magnitude of cognitive impairment.

#### 4.4.3 Biomarkers

We correlated the composite scores of the cognitive domains with several biomarkers that have been suggested to be altered in patients with COVID‐19 (Samprathi & Jayashree, 2020), including: C‐reactive protein (CRP) levels, aspartate aminotransferase (AST), alanine aminotransferase (ALT), lactate dehydrogenase (LDH), creatine kinase (CK), hemoglobin (Hg), platelets, leukocytes, lymphocytes, D‐dimer, ferritin, and interleukin‐6 (IL‐6). Significant correlations were found only for the composite scores of Language with ferritin (*r* = 0.32, *p* = .03) and Attention with CK (*r* = −0.41, *p* = .007).

#### 4.4.4 Affective scores

Correlations between anxiety and depression subscores from the HADS and all domain factors were performed. There were no significant correlations between anxiety subscores and cognitive domains. Depression subscores only showed a significant correlation with Processing Speed (*r* = 0.29, *p* = .02), suggesting a rather weak positive relationship between depression measures and this cognitive domain.

## 5 DISCUSSION

This study was conceived to further characterize the extent of cognitive impairment in post‐COVID‐19 patients with subjective cognitive complaints. A large US survey study found that difficulty concentrating and focusing was experienced by more than 50% of patients, being the fourth most reported long‐term symptom after COVID‐19 (Lambert & Corps, 2020). While cognitive impairments appear to be most pronounced in people who were hospitalized, they have also been observed in non‐hospitalized patients (Hampshire et al., 2021), mild cases of COVID‐19 (Townsend et al., 2020), and in asymptomatic patients (Amalakanti et al., 2021). To investigate how far‐reaching the cognitive sequelae of COVID‐19 are, the present study aimed to (1) provide information on the frequency of deficits in specific cognitive domains and (2) discern the frequency of single‐ and multiple‐domain impairment.

Our results show that all patients in the sample demonstrated cognitive deficits in at least one domain. Because all patients were referred for evaluation due to subjective cognitive complaints, this overall result is not surprising; it does, however, help to confirm these complaints with objective neuropsychological measures.

Considering this study's first aim, the most common domain affected overall was attention (61.9% of the sample). Additionally, attentional deficits were the most frequent deficits in those patients with single‐domain impairment (19.0%), far exceeding isolated impairment in EF, ST/WM, and Language. Of note, impairment in attentional processes in post‐Covid‐19 patients and its potential to compromise other cognitive domains has been pointed out by different studies (Almeria et al., 2020; Zhou et al., 2020). For the present study, attention abilities were indexed by the CPT‐II. Although many CPT‐II subscores revealed large percentages of low scores, most impaired performance was shown on measures pertaining to overall variability of responses (HIT SE, Variability, and HIT SE Block Change; see Figure 1), indicating problems in consistency of responses and inattentiveness. These results provide evidence for pervasive difficulties in sustaining attention throughout a task in this post‐COVID population.

The second most affected domain overall was EF (43% of the sample). However, EF impairment was not commonly isolated, as only 4.8% of the sample demonstrated single‐domain impairment in EF; in most cases, EF was altered in combination with dysfunction in other cognitive domains, possibly explained by its role in control and regulation of proper functioning in other cognitive functions and behaviors (Fuster, 2000). This finding is also in line with results from Miskowiak et al. (2021), who found a positive correlation between executive function deficits and subjective cognitive complaints in a group of COVID‐19 patients assessed four months after hospitalization.

With the high frequency of low average performance in both attentional and executive control tasks, it will be essential to continue to assess this decreased performance with follow‐up neuropsychological evaluations. At this point, we still do not know whether patients experiencing post‐COVID cognitive impairment are likely to revert to average test performance or not. If it is the case that these deficits are not resolved over time, clinical and research professionals will need to formulate specific interventions centered upon general cognitive stimulation or restoring the efficiency of their attention functions.

Regarding our second aim, the results of this study indicate a larger proportion of multi‐domain than single‐domain impairment, with the percentage of patients showing two and more cognitive affected domains at 60.3% and the percentage of patients with single‐domain impairment at 39.7%. Deficits in attention were present in most combinations of multiple‐domain impairment, especially in conjunction with L + LTM and EF. Running correlations between Attention, L + LTM and EF, no significant relationship between L + LTM and Attention was found. However, there was a significant correlation between EF and Attention, suggesting an interdependence between these two domains. This could be explained by shared neural networks in fronto‐subcortical structures that, if damaged, would produce deficits in both domains. Alternatively, impairment in attention abilities could provoke a cascading impact on executive functions. Impairment in memory is likely due to hippocampal dysfunction and exists independently of other types of impairment. To address this question, future studies on cognitive functioning post‐COVID‐19 will need to include neuroimaging and relate brain structural and functional integrity with cognitive performance.

In addition to the associations between cognitive domains, we also examined whether other clinical factors might be linked to cognitive performance. Comparing hospitalized and non‐hospitalized patients in our sample, we found significantly lower performance on the MoCA and in Processing Speed for hospitalized patients. While the significant group differences here are important, what might be more intriguing is that hospitalization did not have a significant effect on test performance in most domains. Given that much of the current literature has focused specifically on COVID patients that were hospitalized (Alnefeesi et al., 2021), our study contributes to emerging evidence (Amalakanti et al., 2021; Lambert & Corps, 2020) that not only do non‐hospitalized patients suffer from post‐COVID cognitive sequelae, but they also may experience similar levels of impairment in domains such as attention, memory, and executive functioning as hospitalized patients.

Finally, we found a strong correlation between ferritin levels during acute illness and the Language composite scores. This correlation could suggest a role for hyper inflammation in neuronal damage underlying language impairment and especially in those language tasks which are timed and involve cognitive flexibility along with optimal frontal functioning. Elevated levels of creatine kinase, associated with decreased muscular function in the context of COVID‐19 (Samprathi & Jayashree, 2021), were negatively correlated with performance on attention measures, indicating an important role for creatine in attention processes. Furthermore, measures of mood alteration did not correlate with attention and EF deficits, indicating that dysfunction in these domains are not associated with levels of depression and anxiety. The only significant correlation found with affective measures was between processing speed and depression; as scores in the domain of processing speed also differed significantly for hospitalized patients, future research should focus on this domain and its interplay with attention and executive functions. Additionally, while our biomarker data was obtained at the time of diagnosis, we suggest that future research should examine the relationship between biomarkers and cognitive deficits using clinical data collected at the time of the neuropsychological assessment. Results from this correlation could provide a more accurate picture of how infection‐related biomarkers relate to individuals’ current cognitive deficits.

We acknowledge that our study has some limitations, namely the absence of a control group. The ideal condition would have been to compare the cognitive performance of our sample with a healthy group of individuals without COVID‐19 or a group of COVID‐19 survivors without subjective cognitive complaints. However, the use of normative data allowed us to draw conclusions about the presence of cognitive deficits and lent support to the validity of our findings.

It is important to keep in mind that, given the absence of a control group without subjective cognitive complaints, our findings cannot be generalized to any patient who was diagnosed with COVID‐19. Nevertheless, we believe that our results, though limited to patients with cognitive complaints, are clinically relevant for neuropsychologists assessing cognition in COVID‐19 survivors.

## 6 CONCLUSION

The findings in the current study begin to shed light on the characteristics of post‐COVID cognitive impairment. Specifically, assessment of patients with subjective cognitive complaints reveals high frequencies of both single‐ and multi‐domain impact that centers upon an intertwined impairment in attention and executive functioning. Additionally, COVID‐19 survivors across the spectrum of disease severity can be left with decreased cognitive function. Clinicians and researchers alike will need to address this by continuing to study post‐COVID patients with comprehensive neuropsychological assessments and applying early interventions to lessen cognitive impairment.

## AUTHOR CONTRIBUTIONS

*Conceptualization*: Carmen García‐Sánchez and Pere Domingo. *Subject recruitment and data curation*: Carmen García‐Sánchez, Catalina Pons, Nicholas Grunden, Juan Antonio Arroyo, Beatriz Gómez‐Anson, Alberto Lleó, Daniel Alcolea, Roberto Belvís, Noemí Morollón, Isabel Mur, and Virginia Pomar. *Methodology*: Marco Calabria, Carmen García‐Sánchez, and Catalina Pons. Data analysis: Marco Calabria. *Project administration*: Carmen García‐Sánchez, Marco Calabria, and Nicholas Grunden. *Supervision*: Pere Domingo. *Writing—original draft*: Marco Calabria, Nicholas Grunden, and Carmen García‐Sánchez. *Writing—review and editing*: Nicholas Grunden, Marco Calabria, Juan Antonio Arroyo, Beatriz Gómez‐Anson, Alberto Lleó, Daniel Alcolea, Roberto Belvís, Noemí Morollón, Isabel Mur, Virginia Pomar, and Pere Domingo.

## CONFLICT OF INTEREST

The authors declare no conflict of interest.

## FUNDING INFORMATION

The research in this study did not receive any funding from public, private, or commercial agencies.

### PEER REVIEW

The peer review history for this article is available at https://publons.com/publon/10.1002/brb3.2508.

## Supporting information



Supporting information
