# Supplementary material for: Neuropsychological deficits in patients with cognitive complaints after COVID‐19
Source: Brain Behav. 2022 Feb 8;12(3):e2508. doi: 10.1002/brb3.2508 (PMC8933779; doi:10.1002/brb3.2508)
Supplement: Supplementary file 1 — Supporting information [file BRB3-12-e2508-s001.docx]

**Supplemental Material – Neuropsychological Tests and Normative Data**

1. *Montreal Cognitive Assessment*^1^ (MoCA)*,* a screening tool for cognitive impairment. It includes subtests in attention and concentration, executive functions, memory, language, visuoconstructional skills, conceptual thinking, calculations, and orientation.
2. *Conners Continuous Performance Test II*^2^ *(CPT-II)*, which measures sustained attention.
3. *Rey's Auditory Verbal Learning Test*^3,4^ *(RAVLT)*, a test of verbal long-term memory and learning;
4. *Rey-Osterrieth Complex Figure Test*^5–7^ (ROCFT), which measures visuoconstructive abilities (copy) and non-verbal long-term memory (delayed recall);
5. *Digit Span Backward and Digit Span Forward*^8–10^*,* which measure short-term and working memory;
6. *Boston Naming Test*^11–13^, that assesses picture naming abilities;
7. *Block Design Test,* a subtest from WAIS-IV^8^, which measures visuoconstructive functions;
8. *Coding Test*, one of two subtests in the Processing Speed Index from WAIS-IV^8^, which measures processing speed by way of visual-motor coordination;
9. *Symbol Search,* the other subtest in the Processing Speed Index from WAIS-IV^8^, which measures processing speed in addition to visual discrimination;
10. *Trail Making Tests*^9,10,14^, which measure the visual attention (part A) and task switching (part B);
11. *Stroop task*^15–17^*,* which measures inhibitory control and verbal interference;
12. *Phonetic and semantic fluency tasks*^18,19^*,* which measure verbal fluency;
13. *15-Objects Test* (15-OT)^20,21^, which measures visual discrimination.

1. Nasreddine ZS, Phillips NA, Bédirian V, et al. The Montreal Cognitive Assessment, MoCA: A brief screening tool for mild cognitive impairment. *J Am Geriatr Soc*. 2005;53(4):695-699. doi:10.1111/j.1532-5415.2005.53221.x

2. Conners CK, Staff MHS, Connelly V, Campbell S, MacLean M, Barnes J. Conners’ continuous performance Test II (CPT II v. 5). *Multi-Health Syst Inc*. 2000;29:175-196.

3. Rey A. L’examen Clinique En Psychologie [The Clinical Psychological Examination] Presses Universitaires de France. *Paris, Fr*. Published online 1964.

4. Schmidt M. *Rey Auditory Verbal Learning Test: RAVLT : A Handbook*. Western Psychological Services; 1996. https://books.google.es/books?id=UOcPRAAACAAJ

5. Osterrieth PA. Le test de copie d’une figure complexe; contribution a l’etude de la perception et de la memoire. *Arch Psychol (Geneve)*. Published online 1944.

6. Palomo R, Casals-Coll M, Sánchez-Benavides G, et al. Estudios normativos españoles en población adulta joven (proyecto NEURONORMA jóvenes): normas para las pruebas Rey-Osterrieth Complex Figure (copia y memoria) y Free and Cued Selective Reminding Test. *Neurología*. 2012;28(4):226-235. doi:10.1016/j.nrl.2012.03.008

7. Peña-Casanova J, Gramunt-Fombuena N, Quiñones-Úbeda S, et al. Spanish multicenter normative studies (NEURONORMA project): Norms for the rey-osterrieth complex figure (copy and memory), and free and cued selective reminding test. *Arch Clin Neuropsychol*. 2009;24(4):371-393. doi:10.1093/arclin/acp041

8. Wechsler D. Escala de Inteligencia Wechsler para adultos IV (Spanish version). Published online 2012.

9. Tamayo F, Casals-Coll M, Sánchez-Benavides G, et al. Spanish normative studies in a young adult population (NEURONORMA young adults project): Guidelines for the span verbal, span visuo-spatial, Letter-Number Sequencing, Trail Making Test and Symbol Digit Modalities Test. *Neurol (English Ed*. 2012;27(6):319-329. doi:10.1016/j.nrleng.2012.07.008

10. Peña-Casanova J, Quiñones-Úbeda S, Quintana-Aparicio M, et al. Spanish multicenter normative studies (NEURONORMA project): Norms for verbal Span, visuospatial Span, letter and number sequencing, trail making test, and symbol digit modalities test. *Arch Clin Neuropsychol*. 2009;24(4):321-341. doi:10.1093/arclin/acp038

11. Goodglass H, Kaplan E, Weintraub S. *Boston Naming Test*. Lea & Febiger Philadelphia, PA; 1983.

12. Aranciva F, Casals-Coll M, Sánchez-Benavides G, et al. Spanish normative studies in a young adult population (NEURONORMA young adults project): Norms for the Boston Naming Test and the Token Test. *Neurol (English Ed*. 2012;27(7):394-399. doi:10.1016/j.nrleng.2011.12.010

13. Peña-Casanova J, Quiñones-Úbeda S, Gramunt-Fombuena N, et al. Spanish multicenter normative studies (NEURONORMA project): Norms for boston naming test and token test. *Arch Clin Neuropsychol*. 2009;24(4):343-354. doi:10.1093/arclin/acp039

14. Bowie CR, Harvey PD. Administration and interpretation of the Trail Making Test. *Nat Protoc*. 2006;1(5):2277-2281. doi:10.1038/nprot.2006.390

15. Golden CJ. Stroop. *Test Color y Palabras Madrid TEA Ediciones*. Published online 1994.

16. Rognoni T, Casals-Coll M, Sánchez-Benavides G, et al. Spanish normative studies in young adults (NEURONORMA young adults project): Norms for Stroop Color–Word Interference and Tower of London-Drexel University tests. *Neurol (English Ed*. 2013;28(2):73-80. doi:10.1016/j.nrleng.2012.02.004

17. Peña-Casanova J, Quiñones-Úbeda S, Gramunt-Fombuena N, et al. Spanish multicenter normative studies (NEURONORMA project): Norms for the stroop color-word interference test and the tower of London-Drexel. *Arch Clin Neuropsychol*. 2009;24(4):413-429. doi:10.1093/arclin/acp043

18. Casals-Coll M, Sánchez-Benavides G, Quintana M, et al. Spanish normative studies in young adults (NEURONORMA young adults project): Norms for verbal fluency tests. *Neurol (English Ed*. 2013;28(1):33-40. doi:10.1016/j.nrleng.2012.02.003

19. Peña-Casanova J, Quiñones-Úbeda S, Gramunt-Fombuena N, et al. Spanish multicenter normative studies (NEURONORMA project): Norms for verbal fluency tests. *Arch Clin Neuropsychol*. 2009;24(4):395-411. doi:10.1093/arclin/acp042

20. Pillon B, Dubois B, Bonnet AM, et al. Cognitive slowing in Parkinson’s disease fails to respond to levodopa treatment: The 15-objects test. *Neurology*. 1989;39(6):762-768. doi:10.1212/wnl.39.6.762

21. Alegret M, Espinosa A, Vinyes-Junqué G, et al. Normative data of a brief neuropsychological battery for Spanish individuals older than 49. *J Clin Exp Neuropsychol*. 2012;34(2):209-219. doi:10.1080/13803395.2011.630652
